# Migration to the US among rural Puerto Ricans who inject drugs: influential factors, sources of support, and challenges for harm reduction interventions

**DOI:** 10.1186/s12889-019-8032-2

**Published:** 2019-12-19

**Authors:** R. Abadie, P. Habecker, C. Gelpi-Acosta, K. Dombrowski

**Affiliations:** 1 Department of Sociology, University of Nebraska-Lincoln, 206 Benton Hall, Lincoln, NE 68588 USA; 2 Social Science Department, LaGuardia Community College, 29-10 Thompson Avenue, Long Island City, NY 11101 USA

**Keywords:** Migration, PWID, Puerto Rico, Continental US, Epidemiological bridge, HIV, Harm reduction

## Abstract

**Background:**

While PWID of Puerto Rican origin have been migrating to the US for decades, the range of factors influencing their migration to the US and the resources they draw on to do so are not well understood. This is particularly true for rural Puerto Rican PWID, and the present study is the first empirical research to document migration patterns among this population. The specificities of their migration raise important challenges that need to be documented in order to implement more effective harm reduction policies at home (Puerto Rico) and abroad (US).

**Methods:**

This paper draws from data obtained employing a modified NHBS survey which was administered to (*N* =296) PWID in four rural municipalities of Puerto Rico with participants 18 years or older. The primary dependent variables for this paper are the number of times a person has lived in the continental US, and if they are planning on moving to the continental US in the future.

**Results:**

Findings suggest that 65% of the sample reported ever lived in the US and that 49% are planning on moving in the future. The number of times living in the US is associated with higher education and older age, but not with self-reported positive HIV or HCV statuses. Planning to move to the US is associated with knowing PWID who have moved or plan to move, negatively associated with age, and is not associated with HIV or HCV status. Around one third of those that lived in the US reported having some sort of support, with the majority receiving support from family sources. No participant received help to enter HIV/HCV treatment.

**Conclusions:**

A multi-region approach to prevention is required to make a dent in curbing HIV/HCV transmission in this population. Understanding PWID migration patterns, risk behaviors, and health care needs in the US is now more important than ever as natural disasters prompted by human-made climate change will only increase in the future, raising demands not only for service providers but also harm reduction policies to cope with an increasing influx of “climate refugees” as PWID move across national borders.

## Background

Migration increases the risk of HIV and hepatitis C (HCV) transmission among people who inject drugs (PWID). When people with HIV and HCV migrate, they carry their viruses with thesm, creating what Rachlis and colleagues have termed an “epidemiological bridge” between the territory they leave and the new one they encounter [42]. Migrant individuals might serve as a bridge to Infectious diseases from high-risk to low-risk individuals and from high-prevalence areas to new locations with low prevalence [57, 58].

In addition, migration disrupts the social networks for habitual injection that PWID rely on to acquire and use drugs, placing them and their co-injecting partners at risk. Migration also introduces PWID into environments that might be culturally difficult to navigate for newcomers, enhancing participation in risk practices [46, 54]. Numerous studies show that migrant PWID engage in different risk behaviors when compared to the native population, including using drugs at a higher frequency and relying on medical treatment less often [19, 50]. Borders facilitate migrant movements, constituting a particular fertile field for the transmission of HIV/HCV among PWID [29, 44]. Studies conducted in the city of Tijuana, on the Mexico-US border, show how migration contributed to increase the risk of HIV/HCV transmission among PWID [39, 56].

Unlike most migrants to the US, Puerto Ricans are US citizens, having first been brought to Hawaii as laborers in the plantation economy more than 100 years ago [52]. Mass migration only started after commercial aviation became more available in the post-war era. Initially, migrants settled mainly in New York City (NYC) and the northeast, with some concentration in Chicago [15, 16]. Decades of migration fueled by economic crises and natural disasters have resulted in more Puerto Ricans living in the continental US than on the island, a movement that only seems to have intensified after Hurricane Maria hit the island in 2017 [34].

Puerto Rican PWID are a part of this migration movement, variously seeking family reunification, economic opportunities, and addiction and/or HIV/HCV treatment options. Migrant PWID who started injecting drugs in Puerto Rico tend to engage in different risk practices in the continental US than their non-migrant PWID counterparts. A study by Gelpí-Acosta and colleagues showed that migrant PWID from Puerto Rico presented higher injection and sexual risk behaviors along with higher HCV prevalence than their US-born PWID counterparts in NYC [21]. Another study of PWID of Puerto Rican origin living in New York and New Jersey found similarly that this group tends to inject frequently and engage in sexual risk behaviors, and that being born in Puerto Rico and purchasing drugs jointly with other users were accurate predictors of injection risks [11, 14]. In addition, family reunification has been identified as an important factor motivating migration among metropolitan Puerto Rican PWID [13].

While these studies make significant contributions towards understanding the relationship between migration and risk behaviors, and shed light on migration motivations among metropolitan Puerto Rican PWID, the range of factors influencing their migration to the US and the resources they draw on to do so are not well understood. This is particularly true for rural Puerto Rican PWID, and the present study is the first empirical research to document migration patterns among this population. The specificities of their migration raise important challenges that need to be documented in order to implement more effective harm reduction policies at home (Puerto Rico) and abroad (US). To begin to address these challenges, this paper examines factors motivating migration and sources of support.

## Methods

### Sample

Surveys were administered to 315 PWID between April and June 2015 in four rural municipalities of Puerto Rico, located approximately 15–30 miles south of San Juan. Eligible participants had to be alert, be 18 years or older, and have injected drugs within the last 30 days. For convenience, interviews were conducted by a trained ethnographer, alongside Puerto Rican staff working out of a storefront in one of the four municipalities, and in close coordination with the region’s only syringe-exchange program (SEP).

### Recruitment

Respondent-driven sampling was used to recruit participants. Respondent-driven sampling is often preferred for hidden and hard-to-reach populations [22]; details about the recruitment process in this study have been published elsewhere [9]. We recruited two PWID (“seeds”) in each of the four municipalities, and after they completed the survey were given three referral coupons to pass out to other PWID from their networks to potentially join the study. Participants could earn an additional $10 for every referral that completed the survey. In a respondent driven framework the interviewer is never aware of the people who may have been given a coupon and refused an interview. In this framework traditional response rate calculations are not applicable. However, the anonymity this provides to potential participants is one of the reasons it is preferred for recruitment of hidden an hard-to-reach populations [23, 26].

### Consent and compensation

All participants gave consent prior to data collection. After completing the questionnaire, participants were paid $25. Ethical clearance was secured from the institutional review boards of the University of Nebraska-Lincoln (IRB# 20131113844FB) and the University of Puerto Rico (IRB# A8480115) prior to data collection.

### Measures

The questionnaire was administered by the interviewer, and was based on the CDC National HIV and Behavioral Surveillance (NHBS) of Injection Drug Users Round 3 Questionnaire version 13 (https://www.cdc.gov/hiv/statistics/systems/nhbs/operations.html). This version of the questionnaire is designed to produce regular estimates of the number of PWID and their behaviors in 23 urban areas in the US, one of which is the city of San Juan, Puerto Rico, which is north of four rural villages surveyed in the present research. In addition, the project provided rapid blood testing for HIV and HCV using INSTI Rapid HIV antibody tests (Biolytical Laboratories) and OraQuick HCV Rapid antibody tests (OraSure Technologies). Participants who tested positive for HIV or HCV antibodies were offered referral and transportation to a primary care doctor for confirmatory testing and link-to-care.

### Measures & analytic strategy

The primary dependent variables for this paper are: *the number of times a person has lived in the continental US* which is a self-reported count, and if they are *planning on moving to the continental US in the future* where a “yes” response is coded as 1, and a “no” response as “0.” We use a multivariable negative binomial regression model to test for associations between the number of times a person has lived in the continental US and the participant’s characteristics. This type of statistical model is appropriate for count outcomes which are over dispersed and do not have a heavy concentration of zeros [30]. Results from the negative binomial regression are discussed in terms of the percent change of the expected number of times a person has lived in the continental US in association with their individual characteristics. Multivariable logistic regression is used to test for associations between participant’s future plans to move to the continental US and their current characteristics. This type of model is appropriate for outcomes that are binary (e.g. can only take the value of 0 or 1) [30]. Results from these models are discussed in terms of percent change in the ratio of the odds that a participant is planning on moving to the continental US and their current characteristics.

In all models, we use the participant’s self-reported HIV and HCV status. Participants could have a self-reported unknown, negative, or positive status. This reflects the results of their more recent test prior to our interview. An unknown report occurs when the participant has never been tested. We use this measure and not the results of the antibody test administered during our interview because we are interested in how their behavior is associated with their own understanding of their HIV or HCV status, and not the results of a test they just completed. For both HIV and HCV we use the self-reported negative category as the reference in the analytical models. The final analytical sample for this paper is 296, with 19 participants removed from the sample due to missing data.

## Results

Our sample is largely male (90.2%), and average 42 years of age (Table 1). The participants are extremely poor, with only 10.5% reporting earning more than $10,000 annually. As many as 47% did not complete high school, while the rest either completed high school (34.5%) or went beyond high school (18.9%). Approximately 47% were single; 31% were either separated, divorced or widowed; and about 22% were cohabiting with or married to another person.

**Table 1:** Descriptive Statistics (*n* = 296)

Variable	% / Mean	Std. Dev.	Min	Max
Female	10%		0	1
Age	41.9	10.1	18	70
Income 10 k+	10%		0	1
Education				
Less than HS	46.96%		0	1
HS	34.46%		0	1
More than HS	18.58%		0	1
Marital Status				
Single	47.3%		0	1
Sep/Div/Widowed	31.1%		0	1
Together	21.6%		0	1
Know other PWID who Have Moved or Plan to Move to Continental US	75.3%		0	1
HIV: Most Recent Status				
Never Tested (Unknown)	14.5%		0	1
Negative	81.4%		0	1
Positive	4.1%		0	1
HCV: Most Recent Status				
Never Tested (Unknown)	23.3%		0	1
Negative	27.0%		0	1
Positive	49.7%		0	1
Previously Lived in the Continental US	64.9%		0	1
Number of Times Lived in the Continental US	1.26	2.00	0	20
Planning on Moving to Continental US	49.0%		0	1

Table 1 shows relevant HIV data: 14.5% of participants reported never being tested, 81.4% had recently tested negative, and only 4.1% had recently tested positive. HCV data include that 23.3% had never been tested, 27% had recently tested negative, and 49.7% had recently tested positive. Migration is a significant phenomenon among these rural PWID, with 75.3% reporting knowing other PWID who either plan to or have already moved to the continental US. Moreover, 64.9% reported *ever living in the continental US*. The average participant had migrated to the continental US 1.26 times, with some participants reporting up to 20 migration events to the US mainland. Additionally, 49% reported “planning to migrate to the continental US” in the future.

Table 2 presents factors associated with the *number of times participants have lived in the continental US*. Those who completed high school had 1.40 (*p* < 0.05) more migration events, and those with education beyond high school had 1.47 (p < 0.05) more migration events than those with less than high school. Whilst educational attainment was associated with migration events, so too was older age. In fact, every year increase in age is associated with a 2% (*p* < 0.01) increase in the expected migration event count. Marital status, income, and knowing other PWID who have already moved or plan to move to the continental US were not associated with migration events. Those who had never been tested for HIV were associated with 47% (*p* < 0.05) lower expected migration events than those with HIV-negative status. We found no difference in the migration counts between those with a negative self-reported HIV status and a positive self-reported HIV status. There were no associations between self-reported HCV status and the number of times a participant has lived in the continental US.

**Table 2:** Multivariable Negative-Binomial Regression of the Number of Times Lived in in the Continental US

Variable	IRR	P	95% CI	
Female	1.11		0.7	1.7
Age	1.02	**	1.0	1.0
Income 10 k+	1.24		0.8	1.9
Education				
Less than HS	Reference			
HS	1.40	*	1.0	1.9
More than HS	1.47	*	1.0	2.1
Marital Status				
Single	Reference			
Sep/Div/Widowed	0.86		0.6	1.2
Together	1.19		0.8	1.7
Know other PWID who Have Moved or Plan to Move to Continental US	0.89		0.6	1.2
HIV: Most Recent Status				
Never Tested (Unknown)	0.53	*	0.3	0.9
Negative	Reference			
Positive	1.04		0.5	2.0
HCV: Most Recent Status				
Never Tested (Unknown)	1.50		1.0	2.3
Negative	Reference			
Positive	1.16		0.8	1.6
Intercept	0.40	*	0.2	0.8
Psuedo-R^2	0.03			
N	296			

* *p* < 0.05, ** *p* < 0.01, *** *p* < 0.001

In Table 3 we present associations with the variable *having plans to migrate to the continental US* in the future. In contrast to the *number of times participants have lived in the continental US*, here education measures were not associated with plans to migrate, and the odds of a participant planning on migrating decreased by 4% (*p* < 0.05) per year of older age. Participants who were separated, divorced, or widowed were associated with 1.97 (*p* < 0.05) times the odds of planning to migrate compared to single participants. In addition, knowing other PWID who had already moved or were planning to move was associated with a 142% (*p* < 0.01) increase in the odds of the participant planning on migrating. Having previously lived in the continental US was also associated with higher odds of planning on migrating in the future, and increase of 71% (p < 0.05). We found no other differences in the odds of planning to migrate to the continental US by any other measure.

**Table 3:** Multivariable Logistic Regression of Plans to Move to the Continental US

Variable	OR	*P*	95% CI	
Female	1.30		0.57	2.96
Age	0.96	**	0.94	0.99
Income 10 k+	1.07		0.48	2.36
Education				
Less than HS	Reference			
HS	0.59		0.34	1.04
More than HS	0.68		0.35	1.34
Marital Status				
Single	Reference			
Sep/Div/Widowed	1.97	*	1.09	3.55
Together	1.59		0.84	3.01
Know other PWID who Have Moved or Plan to Move to Continental US	2.42	**	1.34	4.36
HIV: Most Recent Status				
Never Tested (Unknown)	1.16		0.54	2.50
Negative	Reference			
Positive	0.76		0.22	2.68
HCV: Most Recent Status				
Never Tested (Unknown)	0.64		0.31	1.34
Negative	Reference			
Positive	0.60		0.34	1.08
Previously Lived in Continental US	1.71	*	1.01	2.91
Intercept	2.23		0.60	8.31
Psuedo-R^2	0.07			
N	296			

* *p* < 0.05, ** *p* < 0.01, *** *p* < 0.001

In Table 4 we present the types of organizations and support received by participants to migrate to the continental US in the past. Overall, of the 65% participants with past migration events, only 29.5% (*n* = 57) had received any type of support in Puerto Rico for their move. Most often, participants received support from “family and friends” (61.4%), “other” (22.8%), and “municipal police” (10.5%); the most common type of support came in the form of a “plane ticket” (71.9%), followed by “financial” (36.8%). No participants reported receiving direct connections to HIV or HCV treatment programs.

**Table 4:** Types of Support PWID Had for Moving to Continental US in the Past (n = 57)

Variable	%
Received Any Support For Migration	29.5%
Types of Organizations That Provided Support (Not Mutually Exclusive)	
Pastor/Church/Religious Org.	3.5%
State Police	5.3%
Municipal Police	10.5%
Family/Friends	61.4%
Other	22.8%
Types of Support Received (Not Mutually Exclusive)	
Financial	36.8%
Purchased Plane Ticket	71.9%
Direct Connection to HIV/AIDS Program	0.0%
Direct Connection to HCV Program	0.0%
Direct Connection to Drug Treatment Programs	21.1%
Other	12.3%

## Discussion

This paper explores influential factors of migration to the continental US among rural Puerto Rican PWID. Our findings confirm what other Puerto Rican PWID researchers [7, 8] and Puerto Rican diaspora scholars [17] have found: US-bound migration is a significant phenomenon among rural Puerto Rican PWID, with 65% reporting that they have lived in the US. The study population shares another important trait with the circular migration patterns of Puerto Ricans to the US: many have lived in the US more than once [3, 45]. While being with family or seeking to improve one’s economic chances spur many in the larger Puerto Rican diaspora to migrate to the US [51], there may be other influential factors behind the decision. We found that factors such as higher education and older age were positively correlated with having past migration events. In addition, just like their metropolitan counterparts [36, 48], rural Puerto Rican PWID also record multiple migration events.

Other factors associated with migration include “having lived previously in the US” and “knowing somebody that did.” These factors increase the odds of *planning to migrate to the US*. Arguably, these factors suggest the powerful draw of family and other social networks, which could enhance or limit migration possibilities. The composition of PWID’s social networks could enhance migration plans, as having friends or family in the continental US may facilitate plans to moving to the US. Also, “having lived previously in the US” is arguably indicative of having established social networks abroad. The migration literature is replete with examples of social networks and even immigrant enclaves seamlessly enhancing migration [5, 33, 49]. Interestingly, here we found an inverse relationship to that of past migration events. While education and age were positively associated with “past migration events,” here these factors either not associated with plans to migrate (education) or are negatively associated with planning to migrate (age). It is possible that the latter may be a result of “migration exhaustion,” at the end of a cycle of circular migration between the homeland and the mainland, as older PWID recorded a significantly higher number of past migration events.

The lack of statistical relationship between self-reported HIV/HCV statuses and past or future migration events is noteworthy, and could mean that migration is not associated (or at least not enough) by the presence of infectious diseases. Despite a paucity of health care resources on the island [24, 31, 37], HIV patients are covered by La Reforma, the local version of Medicare/Medicaid, as are those co-infected by HIV and HCV. Despite the fact that HCV treatment is not covered by La Reforma in Puerto Rico and most PWID go untreated since they cannot afford private coverage [1], seeking HCV care in the continental US does not seem to be among their motivations. This finding may appear striking, for many might presume that the dearth of treatment services available in rural Puerto Rico might be a big motivator for migrating to the continental US. Yet, our findings show that even for those who are HCV-positive (a considerably larger subgroup than those with HIV), accessing treatment (in the US) is not at the forefront of their priorities. Other studies have found that HCV does not concern PWID as much as HIV does [2, 47, 55], and this may be a plausible explanation for this finding.

Finally, our findings on the *sources of support* and the *types of support* contain a surprising aspect: most migrants in our study do not have any sources of support (69.5%). This is remarkable given that much of the literature on migrant Puerto Rican PWID traces how family and other types of networks create bridges between the US and PR. Of the 29.5% that lived in the US in the past *and* reported having some sort of support, the majority received support from family. Other sources of support were municipal or state police, and religious organizations. While we only have limited data on these sources of support, it is noteworthy that this is the first time a study has identified these other sources as influential in the migration phenomenon. In addition, the most common form of support (for migration) is a “plane ticket” (around 2/3), usually one-way.

Unlike undocumented PWID from other Latin American origins, Puerto Rican PWID are US citizens, free to move back and forth between the motherland and the mainland. US-citizenship is perhaps the most important migration enhancer, even in the absence of other sources of support for migration. To borrow Deren’s terminology (2007), this “air-bridge” between the US and PR raises important questions about the epidemiological bridge, risk behaviors, and prevention among Puerto Rican PWID enmeshed in this circular migration. A multi-region approach to prevention is required if we were to make a dent in curbing HIV/HCV transmission in this population [12, 29, 38]. While health policies and, in particular, harm-reduction interventions have effectively addressed the link between HIV transmission and migration processes [10, 25, 28, 32, 40, 59], the health challenges posed by migrating PWID have not received the same degree of attention. An exception is the work conducted by Strathdee and colleagues [53, 54] in Tijuana, on the Mexican side of the Mexico-US border, which draws attention to the HIV/HCV transmission risk created by the combined effects of poverty, migration, forced deportation, and a scarcity of health care resources for this vulnerable population in the region. Harm-reduction programs need to recognize that while PWID have traditionally been as a static population in large urban centers—or more recently rural or suburban communities—they also form part of larger migratory flows [35, 43]. Their movement across national borders creates both challenges and opportunities for health policy interventions.

Understanding PWID migration patterns, risk behaviors, and health care needs in the US is now more important than ever. In September 2017 Hurricane Maria devastated the island, raising additional barriers to medication assisted treatment (MAT), syringe exchange providers (SEP), mental health care, and other services that support PWID in rural areas. It also reinforced a migratory process that had gained strength since the economic crisis that started a decade earlier. As PWID join this migratory trajectory to the mainland, more research is needed to understand their migration patterns, health needs, and HIV/HCV risk behaviors. These issues will become increasingly central to the future harm-reduction agenda, as natural disasters prompted by human-made climate change will only increase in the future, raising demands not only for service providers but also public health researchers to cope with an increasing influx of “climate refugees” as PWID move across national borders [4, 6, 18, 20, 27, 41].

## Limitations

One limitation if this study is data about migratory trajectories among PWID from rural Puerto Rico to the continental United States is based not on direct observation but on participants’ recollection. It is possible that responses were affected by recall bias. Subjects might have underestimated the number of times they migrated to the US, or, fail to recall their particular motivations to migrate as well as the resources mobilized in order to migrate. Despite this limitation, we are confident that the data presented represent a significant contribution to an under studied problem.

## Conclusions

Findings suggest that the migration of rural PWID living in Puerto Rico to the continental US is relatively high, with more than half of the sample reporting that they had lived in the US at least at one point in time. Having lived previously in the US and knowing somebody that still did increased the odds that a respondent had lived in the US. Around one-third of those that lived in the US reported having some sort of support, with the majority receiving support from family sources, which reflected goals of family reunification, while one-fifth were linked to a drug treatment program. Finally, there was no relationship between HIV/HCV self-reported status and having lived in the US, and no participant received help to enter HIV/HCV treatment. Migration of PWID in Puerto Rico to the continental US is likely to increase in the future, driven by economic factors and natural disasters, calling for the design of public health policies to address prevention and treatment needs in this population.

## Data Availability

All data generated or analysed during this study are included in this published article [and its supplementary information files].

## Abbreviations

HCV: Hepatitis C Virus
HIV: Human Inmunodeficiency Virus
MAT: Medically Assisted Treatment
NYC: New York City
PWID: People Who Inject Drugs
SEP: Syringe Exchange Providerss
US: United States
